# Effects of Manual Therapy Plus Pain Neuroscience Education with Integrated Motivational Interviewing in Individuals with Chronic Non-Specific Low Back Pain: A Randomized Clinical Trial Study

**DOI:** 10.3390/medicina60040556

**Published:** 2024-03-29

**Authors:** Konstantinos Kasimis, Thomas Apostolou, Ilias Kallistratos, Dimitrios Lytras, Paris Iakovidis

**Affiliations:** Department of Physiotherapy, Faculty of Health Sciences, International Hellenic University, Alexander Campus, P.O. Box 141, 57400 Thessaloniki, Greece; apostolouthomas@hotmail.com (T.A.); elipulse@yahoo.gr (I.K.); lytrasde@gmail.com (D.L.); piakov@ihu.gr (P.I.)

**Keywords:** chronic low back pain, pain neuroscience education, motivational interviewing, manual therapy, physiotherapy

## Abstract

*Background and Objectives*: Chronic non-specific low back pain (CNLBP) persists beyond 12 weeks. Manual therapy recommended for CNLBP demonstrates short-term efficacy. Pain Neuroscience Education (PNE) teaches patients to modify pain perception through explanations, metaphors, and examples, targeting brain re-education. Motivational Interviewing (MI) enhances motivation for behavioral change, steering patients away from ambivalence and uncertainty. These approaches collectively address the multifaceted nature of CNLBP for effective management. The aim of this study was to investigate a manual therapy intervention combined with PNE with MI on pain, pressure pain threshold (PPT), disability, kinesiophobia, catastrophizing, and low back functional ability in individuals experiencing CNLBP. *Materials and Methods*: Sixty adults with CNLBP were randomly divided into three equal groups (each *n* = 20). The first group received manual therapy and PNE with integrated MI (combined therapy group), the second group underwent only manual therapy (manual therapy group), and the third group followed a general exercise program at home (control group). Pain in the last 24 h was assessed using the Numeric Pain Rating Scale (NPRS), functional ability with the Roland–Morris Disability Questionnaire (RMDQ), PPT in the lumbar region through pressure algometry, kinesiophobia with the Tampa Scale for Kinesiophobia (TSK), catastrophizing with the Pain Catastrophizing Scale (PCS), and performance using the Back Performance Scale (BPS) at baseline, in the fourth week, and six months post-intervention. *Results*: Statistically significant differences between the intervention groups and the control group were found in both the fourth-week measurement and the six-month follow-up, as evident in the NPRS and RMDQ scores, as well as in the total values of tested PPTs (*p* < 0.05). Differences were also observed between the two intervention groups, with a statistically greater improvement in the combined therapy group at both time points (fourth week and six-month follow-up) (*p* < 0.05). Regarding the TSK and PCS scores in the fourth week, statistically significant differences were observed between the two intervention groups compared to the control group, as well as between the two intervention groups (*p* < 0.05). However, in the six-month follow-up, statistically significant differences were found only between the combined therapy group and the other two groups, with the combined therapy group showing significant improvements (*p* < 0.05). In relation to BPS, both intervention groups exhibited statistically significant differences compared to the control group in the fourth week, without any significant differences between the two intervention groups. However, in the six-month follow-up, significant differences were noted between the combined therapy group and the other two groups (*p* < 0.05), with combined therapy demonstrating greater improvement. *Conclusions:* The addition of PNE with integrated MI enhanced the positive effects of a manual therapy intervention in all outcome measures. The combination of manual therapy plus PNE with integrated MI appeared to provide greater improvements compared to the isolated application of manual therapy, and these improvements also lasted longer. These short- and long-term positive effects are likely attributed to the combination of PNE with integrated MI, which contributed to increasing the effectiveness of the treatment. Further studies are required to investigate the optimum dosage of manual therapy and PNE with integrated MI in individuals with CNLBP.

## 1. Introduction

Low back pain is defined as pain in the lower back area above the gluteal folds and below the lower margin of the thoracic wall [1,2]. A limited percentage, up to 15%, of individuals with low back pain receive a specific patho-anatomical diagnosis, leaving the majority classified under the category of non-specific [3]. Within this non-specific low-back-pain population, a notable yet significant subset develops chronic and disabling symptoms, referred to as chronic non-specific low back pain (CNLBP). This subgroup tends to utilize a disproportionately high amount of healthcare resources [4]. CNLBP is characterized by the presence of persistent back pain that exceeds a duration of 12 weeks [5]. Clinical guidelines recommend manual therapy as a treatment method for CNLBP, but its benefits are observed to be short-term [6]. Some support it as a standalone treatment, while others advocate for its integration within a combined approach [7,8].

In recent years, CNLBP management techniques have been developed that target the brain’s retraining in pain perception [9]. Pain Neuroscience Education (PNE) is an educational process applied by physiotherapists in which patients are taught how to manage their pain perception. Through this approach, the physiotherapist, utilizing effective communication skills, aims to reduce the patient’s perception of nociceptive stimuli at the level of the central nervous system. The goal of this approach is to elucidate, for patients, the neurobiological processes, pain physiology, nociplastic pain, alterations in the somatosensory system due to pain, and psychosocial manifestations of their pain in a comprehensible manner. The content of this intervention includes explanations, metaphors, examples, and images, which are employed to alter perceptions, rectify misinterpretations, and address maladaptive thoughts linked to pain [10,11].

In contrast to manual therapy, where the physiotherapist solely focuses on tissue pathology, PNE aims to reconceptualize pain by teaching patients more about the neurobiological and neurophysiological processes involved in their pain experiences [9]. However, recent evidence suggests that adding PNE to a manual therapy intervention further increases the hypoalgesic effect and improves short-term outcomes related to kinesiophobia and catastrophizing [12,13]. Some authors suggest that these benefits are likely due to enhancing patient expectations while contributing to the refreshing or sharpening of body schema maps within the brain [9].

Motivational Interviewing (MI) is a communication strategy revolving around patients to motivate them to change their behaviors, guiding patients away from ambivalence and uncertainty [14,15]. PNE and MI represent distinct yet complementary approaches in healthcare, particularly in the realm of chronic pain management. Some authors suggest that combining PNE with MI can improve outcomes with greater and more long-term results [16].

The aim of this clinical study is to examine the short-term and long-term effects of the addition of PNE with integrated MI in a manual therapy program on pain intensity, pressure pain threshold (PPT), low back functional ability, disability, kinesiophobia, and catastrophizing among individuals suffering from CNLBP. The conduction of this research was based on the hypothesis that integrating MI into PNE further enhances the positive effects of the manual therapy intervention compared to the isolated implementation of manual therapy and also makes these improvements last longer.

## 2. Materials and Methods

### 2.1. Study Design

Our assessor-blinded randomized clinical trial (clinicaltrials.gov number: NCT05928975) was carried out under the supervision of the Department of Physiotherapy of the International Hellenic University from January to September 2023, adhering to the consolidated standards of reporting trials (CONSORT) extension for pragmatic clinical trials. Ethical approval was obtained from the Ethics Committee of the Department of Physiotherapy at the International Hellenic University (No. EC-08/2022). Sixty individuals with CNLBP were recruited from three outpatient physiotherapy clinics and were randomly allocated into two intervention groups and one control group (each *n* = 20). Allocation concealment was achieved by an independent researcher using Research Randomizer software (version 4) [17] for participant distribution. The randomization process, conducted in small groups of three, employed block randomization with a 1:1 ratio in each of the three groups. None of the healthcare providers or participants were blind to the study’s aims.

### 2.2. Participants

Inclusion criteria for the study were (1) symptoms lasting at least 12 weeks, (2) self-reported pain intensity of 3 or higher using the numeric pain rating scale (NPRS), (3) age ranging from 25 to 60 years, and (4) providing written consent to participate in the study. Exclusion criteria were (1) history of neuropathic pain extending along the lower limb due to nerve root compression, (2) previous spinal surgery, (3) history of spinal trauma or fracture, (4) presence of cancer, (5) severe osteoporosis, (6) spondylo-arthropathy, (7) spondylolisthesis, (8) systemic inflammatory disease, (9) illiteracy, (10) diagnosed neurodegenerative diseases (e.g., Parkinson’s), (11) epilepsy, and (12) history of psychiatric disorders.

### 2.3. Measurements

Outcomes were measured at baseline, in the fourth week, and six months post-intervention. All outcome measurements were considered primary.

#### 2.3.1. Pain with the Numeric Pain Rating Scale (NPRS)

The NPRS is an eleven-point pain scale ranging from zero to 10. On the scale, zero is labeled as “no pain at all” at the left end, and 10 is labeled as “worst possible pain” at the right end. Consequently, a higher value indicates more intense pain [18]. The individual is requested to select an integer that best represents the severity of their pain. The NPRS is widely employed for pain assessment in both clinical practice and research, demonstrating robust test–retest reliability and sound conceptual construct validity [18,19]. The minimal clinically important difference (MCID) in individuals with CNLBP was indicated to be 2.5 points [20].

#### 2.3.2. Low Back Pain Disability with the Roland-Morris Disability Questionnaire (RMDQ)

Participants’ disability resulting from CNLBP was assessed using the Greek version of the Roland–Morris questionnaire (RMDQ). The RMDQ is a 24-item questionnaire concerning daily activities that individuals commonly find challenging due to lower back pain [21,22]. Each affirmative response accumulates one point, and the total score is determined by adding up all the points. Hence, a higher score indicates more significant restrictions. The Greek version of the questionnaire exhibits satisfactory reliability and validity (ICC: 0.44–0.92) [23]. According to Ostelo and de Vet [20], MCID was indicated to be 3.5 points for all types of low back pain.

#### 2.3.3. Pressure Pain Threshold (PPT) Using Pressure Algometry

The pressure pain threshold (PPT) is defined as the minimum pressure required to elicit pain. PPT assessments were performed bilaterally in the quadratus lumborum muscle, and paravertebrally in the L4-L5 intervertebral space using a digital algometer (Wagner FPX 25 Digital Algometer; Wagner Instruments, Greenwich, CT, USA). The metal rod of the algometer was positioned vertically on the site, and the examiner applied pressure gradually, increasing at a rate of 10 N/s. PPT values were calculated in N/cm^2^. The measurement protocol followed the instructions outlined by Imamura et al. [24,25]. The reliability of PPT assessment in the low back region is excellent, with ICC values ranging from 0.86 to 0.99 [26]. An MCID was considered an increase in PPT value bigger than 15% [27].

#### 2.3.4. Kinesiophobia Using the Tampa Scale for Kinesiophobia (TSK)

Kinesiophobia was assessed with the Tampa Scale for Kinesiophobia (TSK), a specialized 17-item questionnaire for assessing fear of movement and re-injury. The questionnaire involves factors related to injury and re-injury, as well as fear-avoidance behaviors in daily activities. Participants provide ratings for each item on a 4-point Likert-type scale, ranging from 1 (strongly disagree) to 4 (strongly agree). The total TSK score falls within the 17 to 68 points range, with higher scores indicating a heightened level of kinesiophobia. Scores of 37 or less are suggestive of a low fear of movement and scores greater than 37 indicate a high fear of movement [28]. The TSK demonstrates a strong level of internal consistency across all items and exhibits a positive correlation with associated measures of fear avoidance, pain catastrophizing, and pain-related disability [29]. The Greek version of the TSK exhibits satisfactory internal consistency and test–retest reliability (Cronbach’s a = 0.74 and ICC = 0.78) [30]. The MCID for the TSK is documented to be a reduction greater than 11% in the total score [31].

#### 2.3.5. Catastrophizing with Pain-Catastrophizing-Scale (PCS)

The Pain Catastrophizing Scale (PCS) is a commonly used self-report questionnaire created to gauge the level to which individuals indulge in catastrophic thinking during pain episodes [32,33]. Comprising 13 items, each details diverse thoughts and emotions that individuals may have when in pain. Participants assess the intensity of each statement on a 5-point Likert-type scale, ranging from “not at all” to “all the time”. A total score ranges from 0 to 52. A higher score corresponds to a higher level of catastrophic thinking. The scale includes three primary dimensions of catastrophizing: rumination, magnification, and helplessness. The MCID for the PCS has been found to range from 3.2 to 4.5 points [34]. The Greek version of PCS exhibits high levels of test–retest reliability (ICC = 0.85) and acceptable internal consistency (Cronbach’s a = 0.80) [35].

#### 2.3.6. Back Performance with Back Performance Scale (BPS)

Back performance was evaluated utilizing the Back Performance Scale (BPS) [36]. The BPS incorporates five tests of trunk mobility (the sock test, pick-up test, roll-up test, fingertip-to-floor test, and lift test). Each test is assigned a score from zero to three according to the observed level of physical performance, and the total score ranges from zero to fifteen points. A high score indicates poor performance. The BPS assesses a facet of physical performance that holds clinical significance for individuals experiencing back pain [36]. The BPS appears to be a reliable and valid outcome measure of activity limitation [37].

### 2.4. Experimental Protocols

#### 2.4.1. Manual Therapy Group

Participants in the manual therapy group received a manual therapy protocol that involved the implementation of a series of different manual techniques. The treatment approach focused on symptom management, guided by the clinical judgment of the attending physiotherapist. The program involved spinal mobilization/manipulation, soft tissue mobilization, supervised exercises, and neural mobilization, without any electrophysical properties. Each participant was scheduled to undergo 10 30-min manual therapy sessions over a span of 4 weeks.

#### 2.4.2. Combined Therapy Group

Participants in this group received the same manual therapy approach as the first group, enhanced by PNE with integrated MI sessions. The PNE with integrated MI intervention consisted of four personalized 30-min sessions, conducted weekly over the four-week intervention period after the manual therapy sessions. The implementation and content of PNE were informed by published works [38,39]. Comprehensive explanations and discussions on all crucial aspects of pain neuroscience were provided using metaphors, examples, and pictures. Educational material was distributed after the initial session to bolster understanding [40]. MI was integrated with PNE delivery according to the suggestions of Nijs et al. [16]. The detailed content of PNE with integrated MI is outlined in Table 1.

#### 2.4.3. Control Group

Participants in the control group were given written instructions for carrying out general exercises at home. These exercises included mild stretching exercises specifically focusing on the muscles of the lower back, along with positions to alleviate discomfort and simple breathing exercises.

### 2.5. Sample Size Calculation

A minimum total sample size of 45 subjects was determined through a priori power analysis (G*Power 3.0.10). In establishing this calculation, a power (1-β) of 95% and the ability to detect a difference with Cohen’s f effect size of 0.5 were considered [41]. The alpha level for all tests was set at 0.05. To account for the six-month follow-up after the intervention, an additional 15% was incorporated into the calculated sample size. Consequently, the projected number of participants to be recruited for this study was 60, based on the aforementioned sample size calculation.

### 2.6. Statistical Analysis

Statistical analysis was carried out using SPSS Statistics for Windows, Version 25.0 (SPSS Inc., Chicago, IL, USA). The Shapiro–Wilk test, Q-Q plots, and P-P plots were utilized to assess normal distribution. Descriptive and frequency analyses were employed to present demographic characteristics. Continuous variables were reported using mean values and standard deviation. Similarly, quality variables were presented using frequencies and percentages. Between-group differences in demographic characteristics were assessed using one-way analysis of variance (ANOVA) for continuous variables and the chi-square test for categorical variables. A two-way ANOVA with repeated measures was used to examine the interaction effect of the “Group” and “Time of measurement”. The “Group” factor was examined across three levels: the manual therapy group, the manual therapy plus PNE with integrated MI (combined therapy) group, and the control group. Simultaneously, the “Time of assessment” factor was evaluated at three levels (baseline, fourth week, and six-month follow-up). If the interaction was statistically significant, the simple main effects were determined using Tukey’s post-hoc test (HSD). An intention-to-treat analysis was performed on all participants within their assigned groups to ensure randomization and address potential dropout effects. In cases of dropout, values from the preceding time point were substituted. The level of significance was set at *p* < 0.05.

## 3. Results

**Figure 1:**
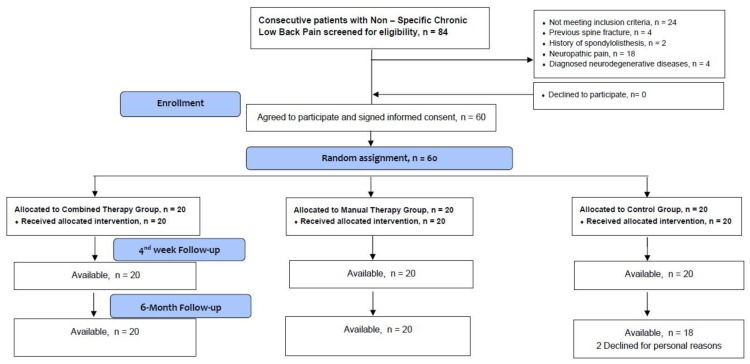
CONSORT flow diagram of patient recruitment.

From January 2023 to March 2023, 84 individuals underwent eligibility screening. Only 60 individuals (71.4%) fulfilled the inclusion criteria and were randomly allocated to one of the three groups (interventions or control) (Figure 1). Two individuals from the control group withdrew from the study due to personal reasons during the six-month follow-up period. No other participant prematurely withdrew from the study. There were no missing sessions or measurement visits during the intervention period. Additionally, none of the participants reported any adverse effects during the implementation of the treatment protocols. The demographic characteristics of participants per group are summarized in Table 2.

### 3.1. NPRS Score

The overall group-by-time interaction from the 2-way ANOVA for the NPRS variable was statistically significant (*p* < 0.001) (Table 3). From pairwise comparisons, differences were observed in the fourth week between the two intervention groups “combined therapy” and “manual therapy” (*p* < 0.05), and between each intervention group and the control group (“combined therapy“ versus “control” and “manual therapy” versus “control”) (*p* < 0.001). These differences persisted at the six-month follow-up measurement.

### 3.2. RMDQ Score

The overall group-by-time interaction for the 2-way ANOVA regarding the RMDQ score was statistically significant (*p* < 0.001) (Table 3). Pairwise comparisons revealed significant differences between the two intervention groups “combined therapy” and “manual therapy” (*p* < 0.05), and between each intervention group and the control group (“combined therapy” versus “control” and “manual therapy” versus “control”) (*p* < 0.001) in the fourth week, while these differences remained unchanged during the six-month follow-up.

### 3.3. TSK Score

Concerning the TSK score results, the overall group-by-time interaction for the 2-way ANOVA was statistically significant (*p* < 0.001) (Table 3). Results from multiple comparisons post-hoc testing demonstrated a substantial difference between groups in the fourth week between the two intervention groups “combined therapy” and “manual therapy” (*p* < 0.05), as well as between each intervention group and the control group (“combined therapy” versus “control” and “manual therapy” versus “control”) (*p* < 0.05). In the six-month follow-up, between-group differences were observed between the two intervention groups “combined therapy” and “manual therapy” (*p* < 0.05) and between the “combined therapy” group and “control” group (*p* < 0.001).

### 3.4. PCS Score

Concerning the PCS score, the overall group-by-time interaction from the 2-way ANOVA was statistically significant (*p* < 0.001) (Table 3). Results from multiple comparisons post-hoc testing showed significant differences between the two intervention groups “combined therapy” and “manual therapy” (*p* < 0.05) and between each intervention group and the control group (“combined therapy” versus “control” and “manual therapy” versus “control”) in the fourth week (*p* < 0.001). In the six-month follow-up, differences were observed both between the two intervention groups “combined therapy” and “manual therapy” (*p* < 0.001) and between the “combined therapy” and “control” groups (“combined therapy” versus “control”) (*p* < 0.001).

### 3.5. BPS Score

The overall group-by-time interaction from the 2-way ANOVA regarding the BPS score was statistically significant (*p* < 0.001) (Table 3). Results from multiple comparisons post-hoc testing indicated between-group differences between each intervention group and the control group (“combined therapy” versus “control” and “manual therapy” versus “control”) (*p* < 0.05) in the fourth week. In the six-month follow-up, differences between groups were observed both between the two intervention groups “combined therapy” and “manual therapy” (*p* < 0.001) and between the “combined therapy” and “control” groups (“combined therapy” versus “control”) (*p* < 0.001).

### 3.6. PPT of L4-L5 Paraspinal Intervertebral Space

Regarding the L4-L5 paraspinal intervertebral space PPTs, the overall group-by-time interaction from the 2-way ANOVA was statistically significant (*p* < 0.001) (Table 3). Results from multiple comparisons post-hoc testing showed significant differences in the fourth week between the two intervention groups “combined therapy” and “manual therapy” (*p* < 0.05), and between each intervention group and the control group (“combined therapy” versus “control” and “manual therapy” versus “control”) (*p* < 0.001). In the six-month follow-up, differences between groups were evident both between each intervention group and the control group (“combined therapy” versus “control” and “manual therapy” versus “control”) (*p* < 0.05) and between the two intervention groups “combined therapy” and “manual therapy” (*p* < 0.05).

### 3.7. Quadratus Lumborum Muscle PPT

Concerning the quadratus lumborum muscle PPTs on both sides (right and left), the results indicate that the overall group-by-time interaction from the 2-way ANOVA was statistically significant (*p* < 0.001) (Table 3). Results from multiple comparisons post-hoc testing (Tukey HDS) demonstrated significant differences in the fourth week between the two intervention groups “combined therapy” and “manual therapy” (*p* < 0.05), and between each intervention group and the control group (“combined therapy” versus “control” and “manual therapy” versus “control”) (*p* < 0.05). At the six-month follow-up time point, differences between groups were evident both among each intervention group and the control group (“combined therapy” versus “control” and “manual therapy” versus “control”) (*p* < 0.05), and between the two intervention groups “combined therapy” and “manual therapy” (*p* < 0.05).

## 4. Discussion

The objective of this clinical study was to examine the effectiveness of a manual therapy protocol in conjunction with the provision of PNE with integrated MI. Compared to other studies that similarly applied combined manual therapy and PNE protocols in individuals with CNLBP [12,13,42,43], the innovation of our study was the integration of the MI component in PNE. We hypothesized that the addition of MI to PNE in a manual therapy program could increase the positive effects and provide more long-term results related to the application of manual therapy alone. This hypothesis is grounded in the notion that combining educational and cognitive therapy approaches within a behavioral framework may help participants manage their pain more effectively. Some authors suggest that the reconceptualization of pain and the reduced threat to the brain that PNE provides may be more effective and individualized when combined with MI [16]. The patient-centered communication style inherent in MI may synergize with PNE, assisting individuals in making behavioral changes related to pain. Increased awareness of the multifaceted nature of pain and behavioral adaptations linked to pain management could improve treatment adherence and amplify receptiveness, acceptance, and engagement, leading to more substantial and enduring effects [16,44].

From the results of our research on pain and disability, it emerged that the combination of manual therapy and PNE with integrated MI was more effective compared to the isolated application of manual therapy. Indeed, participants in the combined therapy group demonstrated more effective improvement in all outcomes related to pain intensity and low back pain disability compared to both the participants in the control group and those in the manual therapy group at both time points (fourth week and six-month follow-up), indicating short- and long-term effects. More specifically, concerning pain intensity, the NPRS score significantly improved in both intervention groups compared to the control group in the fourth week, and these differences remained statistically significant during the six-month follow-up (Table 3). Both intervention groups exhibited a notable decrease in the NPRS score in the fourth week compared to baseline (3.6 points, corresponding to a 61.01% reduction in the combined therapy group, and 2.7 points, corresponding to a 45.0% reduction in the manual therapy group). These differences were also clinically significant for both intervention groups (a decrease greater than 2.5 points) [20].

However, statistically significant differences were observed in the fourth week between the two intervention groups (“combined therapy” versus “manual therapy” group), with the combined therapy group showing further pain reduction compared to the manual therapy group (Table 3). It is also noteworthy that the changes between the two intervention groups remained statistically significant during the six-month follow-up, indicating that the impact of the manual therapy and PNE combination with integrated MI persisted even six months after the intervention. Moreover, the maintenance of the difference in the combined therapy group during the six-month follow-up has clinical significance, as only participants in this group retained a difference greater than 2.5 points compared to the initial measurement (3.35 points). The changes we found in the NPRS score at the six-month follow-up are greater compared to another study in which only the combination of manual therapy and PNE (without MI) was applied [43], where the reduction in NPRS during the six-month follow-up compared to the initial measurement was only 0.81 points.

Regarding the PPT results, statistically significant differences were observed in all measured points (L4-L5 paraspinal intervertebral space PPT right and left and quadratus lumborum right and left) between the two intervention groups and the control group in the fourth week, which remained statistically significant during the six-month follow-up. Differences were also found between the two intervention groups (“combined therapy” versus “manual therapy”), with the “combined therapy” group showing statistically significant improvements in all PPT measurements both in the fourth week and during the six-month follow-up (Table 3). It is worth noting that the improvements in both intervention groups compared to the baseline measurement were clinically significant, as participants in both groups exhibited increases in all PPTs greater than 15%, which constitutes an MCID according to Voogt et al. [27]. Our research findings partially align with those of Malfliet et al. [45], who also observed statistically significant improvements in PPT values by implementing PNE combined with cognition-targeted motor control training in individuals with spinal pain. Additionally, our results are consistent with the findings of Bodes Pardo et al. [46], who identified statistically significant improvements in PPT values in patients with CNLBP by applying PNE within a therapeutic exercise program.

The results regarding the RMDQ score revealed a statistically significant improvement in both intervention groups compared to the control group in the fourth week (5.95 points, corresponding to a 56.13% reduction in the combined therapy group, and 2.95, corresponding to a 29.64% reduction in the manual therapy group). These differences remained statistically significant during the six-month follow-up (Table 3). Notably, significant differences were observed between the two intervention groups (“combined therapy” versus “manual therapy” group) in the fourth week, with the combined therapy group showing a statistically greater reduction in the RMDQ score compared to the manual therapy group (Table 3). This may be attributed to a further decrease in pain intensity experienced by the combined therapy group compared to the manual therapy group, as reflected in the changes in the NPRS and PPT scores. Importantly, the statistically significant difference between the two intervention groups persisted during the six-month follow-up, indicating that the combination of manual therapy and PNE with integrated MI appeared more effective in the long term compared to the isolated application of manual therapy. Additionally, it is crucial to highlight that the improvement observed in the combined therapy participants is clinically significant, as only this group demonstrated an improvement greater than 3.5 points, which aligns with the MCID criteria according to Ostelo and de Vet [20]. Our study results are partly consistent with those of Tavarez et al. [43], who, using a combined manual therapy with the PNE protocol, also observed long-term improvements in low back pain disability six months post-intervention. However, it is important to note that they utilized a different assessment tool (the Oswestry Disability Index), making a direct comparison of results challenging.

The fact that both intervention groups showed significant improvements in pain intensity and low back pain disability compared to the control group highlights, in our opinion, the effectiveness of manual therapy in reducing pain. Research indicates that the hypoalgesic effect of manual therapy results from neurophysiological modifications in the stimulation of alpha motor neurons and immune response systems and a rise in endorphins and serotonin in the bloodstream, occurring across the nervous system [47]. However, the additional improvement noted in the overall variables related to pain intensity and low back pain disability in participants of the combined therapy group compared to those in the manual therapy and control groups may be attributed to the inclusion of PNE with integrated MI in the manual therapy intervention. PNE, as well as MI, are both established interventions in chronic pain management, extensively studied for short-term effectiveness [10,15]. PNE uses contemporary pain science to explain the biopsychosocial aspects of chronic pain, optimizing patients’ beliefs for coping. On the other hand, MI employs a patient-centered approach to enhance motivation for change, guiding away from indecision. Conceptually, PNE and MI complement with non-overlapping effects. MI improves awareness, potentially enhancing treatment adherence, while PNE elevates pain knowledge, modifies beliefs, and fosters a willingness to explore psychological factors associated with pain. [16]. One possible explanation could be that the integration of MI into PNE assisted participants in finding an optimal way to develop a heightened understanding of the source and perception of pain, effectively managing their negative emotions, and ultimately enhancing their effectiveness in the extended-term management of their symptoms. Furthermore, the fact that all the improvements persisted during the six-month follow-up corroborates the assumptions of Nijs et al. [16], according to whom combining PNE with MI might lead to improved outcomes with larger and longer-lasting benefits.

As for the results concerning kinesiophobia and catastrophizing, the following observations were noted based on our research.

Regarding the TSK results, statistically significant differences emerged between the two intervention groups and the control group in the fourth week (“combined therapy“ versus. control and “manual therapy” versus control) and between the two intervention groups (“combined therapy” versus “manual therapy”) (Table 3). This means that both manual therapy alone and the combination of manual therapy and PNE with integrated MI effectively reduced levels of kinesiophobia over the four-week intervention period. Moreover, this improvement was clinically significant, as both intervention groups demonstrated changes greater than 11%, which constitutes the MCID for the TSK score [31]. However, this reduction was much larger (almost double) in participants in the combined therapy group, with a statistically significant difference (12.15 points, corresponding to a 27.30% reduction in the combined therapy group, and 6.75, corresponding to a 15.69% reduction in the manual therapy group). This fact demonstrates the superiority of the combination of manual therapy and PNE with integrated MI compared to manual therapy alone. However, during the six-month follow-up, differences were observed only between the “combined therapy” group and the control group. A possible interpretation is that participants in the combined therapy group maintained a reduction in kinesiophobia for a longer period compared to participants in the manual therapy group. 

Similarly, we observed significant results in the PCS score. The statistically significant differences noted in the fourth week between the two intervention groups and the control group, as well as the differences between the two intervention groups themselves, indicate that both the isolated application of manual therapy and the combination of manual therapy plus PNE with integrated MI effectively improved levels of catastrophizing after four weeks of intervention. This improvement, beyond being statistically significant, also holds clinical significance, as both intervention groups demonstrated improvements greater than 4.5 units (compared to baseline values), which represents the MCID for the PCS score [34]. However, the fact that participants in the combined therapy group showed much greater improvement in the fourth week compared to those in the manual therapy group (11.95 points, corresponding to a 35.58% reduction in the combined therapy group, and 6.45, corresponding to a 19.48% reduction in the manual therapy group), means that the combination of manual therapy and PNE with integrated MI more effectively reduced levels of catastrophizing in participants compared to the isolated application of manual therapy. However, during the six-month follow-up, statistical differences were noted solely between the combined therapy group and the control group. One potential interpretation is that participants in the combined therapy group sustained a decrease in catastrophizing tendencies for a more extended period compared to those in the manual therapy group.

One possible interpretation of the TSK and PCS results is that the significant improvements in TSK and PCS scores observed in the manual therapy group during the fourth week may be attributed to the hypoalgesic effect of manual therapy experienced by this group. Therefore, it can be stated that the manual therapy program we implemented, by reducing pain, also decreased levels of kinesiophobia and catastrophizing among participants. However, the fact that these improvements were not sustained during the six-month follow-up may be due to the participants not changing their behavior towards pain beyond experiencing hypoalgesia. In fact, some evidence suggests that manual therapy techniques alone do not demonstrate substantial variations in mitigating kinesiophobia and catastrophizing, contributing to a decrease in local pressure pain thresholds among individuals with musculoskeletal pain [27,48]. Hands-on techniques primarily focus on the symptomatic area (i.e., local anatomical structures), lacking a direct impact on more intricate mechanisms related to chronic pain, such as brain-level sensitization. Additionally, they do not directly influence the psychological and socioeconomic factors linked to the intensity and duration of symptoms [49].

In contrast, participants in the combined therapy group attribute their greater improvement in the fourth week not only to the significant reduction in pain but also to the reconceptualization of pain and the diminished threat to the brain achieved through PNE with integrated MI. Furthermore, the maintenance of positive effects during the six-month follow-up is likely due to participants adopting changes in their behavior towards pain and becoming more capable of managing their symptoms in the long term. Our research results align with those of Saracoglou et al. [12], who found further improvement in kinesiophobia by adding PNE to a manual therapy program. The researchers attributed this effect to the Fear Avoidance Model applied as PNE [50], which effectively reduced levels of kinesiophobia by explaining anxiety, fear, avoidance due to fear, and their consequences for pain experience. Our research results also align with the findings of Song et al. [13], who identified a significant reduction in the TSK and PCS scores by adding PNE to a four-week manual therapy program for patients with CNLBP. However, it is worth noting that the improvements identified in our study were greater for both scores (TSK and PCS) compared to this research. This difference may be attributed to the integration of MI into PNE. We believe that by using MI strategies, such as change talk (elicit–provide–elicit), we further increased patient receptiveness, acceptance, and engagement, leading to improved treatment adherence. Additionally, the integration of MI into PNE induced patient-centered communication, enhancing the treatment’s overall effectiveness.

Regarding the performance of the BPS, statistically significant differences were observed only between the combined therapy group and the control group in the fourth week, which remained statistically significant during the six-month follow-up. Differences between the intervention groups were also noted, but only during the six-month follow-up. This seems normal since participants in the combined therapy group experienced lower levels of pain, low back pain disability, kinesiophobia, and catastrophizing. It appears that the improvement in the BPS score during the fourth week in the manual therapy group (even if not statistically significant) was due to the hypoalgesic effect of manual therapy. The fact that during the six-month follow-up, the difference compared to the combined therapy group was diminished, may be attributed to the absence of changes in behavior and the way the participants perceived pain, unlike the manual therapy group. Our findings disagree with Saracoglou et al. [12] who, while studying the effects of adding a PNE protocol to a four-week manual therapy program (similar to ours), did not find further improvement in the BPS score.

Our study encountered various limitations. The inability to blind both participants and care providers to the study’s objectives posed a potential threat. Additionally, the research was constrained by a small number of participants and a relatively short follow-up period, which constituted significant drawbacks.

## 5. Conclusions

The addition of PNE with integrated MI further improved the positive effects of a manual therapy program on pain intensity, pain thresholds, disability, kinesiophobia, catastrophizing, and back performance. The combination of manual therapy plus PNE with integrated MI appeared to result in greater improvements compared to the isolated application of manual therapy, and these improvements also lasted longer. These short- and long-term positive effects are likely attributed to behavioral adaptations and reconceptualization of pain derived from PNE with integrated MI, which increased treatment effectiveness. Further studies are required to investigate the optimum dosage of manual therapy and PNE with integrated MI in individuals with CNLBP.

## Figures and Tables

**Table 1:** Content of PNE with integrated MI.

**Session 1**	Thorough interview, including biopsychosocial intake Obtained explicit consent from the patient (shared-decision format) Establishment of a therapeutic alliance between the patient and the clinician Identification of the patient’s issues to be addressed during the subsequent tailored PNE/MI sessions Introductory PNE session (using the alarm system metaphor)
**Sessions 2–4**	At the beginning of each follow-up session, there was a quick review of the previous session’s information, and patient questions were answered PNE content was tailored to the patient’s needs and pain beliefs/perceptions, as identified during Session 1 PNE included topics such as pain neurophysiology, nociception and nociceptive pathways, peripheral and central sensitization, the nervous system plasticity, and psychosocial factors and beliefs contributing to pain PNE topics discussed by eliciting change talk (elicit-provide-elicit approach). Specifically, patients were invited to express their thoughts about a PNE topic about to be discussed, then permission was sought by the clinician to provide information and current knowledge about that topic, and finally, the patient was asked to express their thoughts on this new information.

**Table 2:** Demographic data of each group. Continuous variables (height, weight, BMI, and duration of pain) are expressed as Mean ± Standard Deviation (SD). Categorical variables are expressed as percentages.

Demographics	Group 1 (Combined Therapy)	Group 2 (Manual Therapy)	Group 3 (Control)	*p*-Value (between Groups)
Age (years)	46.60 (8.89)	45.50 (9.58)	45.90 (9.38)	0.93
Sex (Men/Women)	45% (*n* = 9)/55% (*n* = 11)	40% (*n* = 8)/60% (*n* = 12)	40% (*n* = 8)/60% (*n* = 12)	0.42
Height (cm)	166.45 (9.33)	167.30 (7.57)	164.50 (7.87)	0.55
Weight (kg)	70.40 (12.31)	72.5 (8.73)	68.75 (11.49)	0.55
BMI (kg/m^2^)	25.17 (0.46)	25.79 (0.24)	25.33 (0.42)	0.50
Duration of pain (months)	18.35 (9.17)	18.65 (7.39)	18.60 (10.70)	0.85

Combined therapy = Manual Therapy plus Pain Neuroscience Education with integrated Motivational Interviewing; BMI = Body Mass Index.

**Table 3:** Results of the 2-way ANOVA analysis and post hoc tests with corresponding *p*-values (*p*-values interaction and *p*-values between groups) at each time point.

	Baseline	4th Week	6-Month Follow-Up
**NPRS score (SD)**			
Group 1 (Combined Therapy)	5.90 (1.16)	2.30 (1.26)	2.55 (1.19)
Group 2 (Manual Therapy)	6.00 (1.29)	3.30 (1.08)	3.65 (1.26)
Group 3 (Control)	6.15 (1.13)	5.50 (1.14)	5.45 (1.43)
*p*-Value (Interaction)	<0.001		
*p*-Value (between groups)	*p* ^a,b,c^ > 0.05	*p* ^a,b,c,^*	*p* ^a,b,c,^*
**RMDQ score (SD)**			
Group 1 (Combined Therapy)	10.60 (3.10)	4.65 (1.42)	5.70 (1.52)
Group 2 (Manual Therapy)	9.95 (3.41)	7.00 (2.49)	8.00 (2.36)
Group 3 (Control)	10.50 (2.43)	8.75 (2.46)	10.35 (2.58)
*p*-Value (Interaction)	<0.001		
*p*-Value (between groups)	*p* ^a,b,c^ > 0.05	*p* ^a,b,c,^*	*p* ^a,b,c,^*
**TSK score (SD)**			
Group 1 (Combined Therapy)	44.50 (4.68)	32.35 (3.32)	34.00 (2.79)
Group 2 (Manual Therapy)	43.00 (5.32)	36.75 (4.76)	38.25 (4.77)
Group 3 (Control)	42.25 (4.27)	40.70 (3.84)	41.10 (4.07)
*p*-Value (Interaction)	<0.001		
*p*-Value (between groups)	*p* ^a,b,c^ > 0.05	*p* ^a,b,c,^*	*p* ^a,b,^*
**PCS score (SD)**			
Group 1 (Combined Therapy)	34.55 (5.12)	22.60 (3.21)	21.30 (2.88)
Group 2 (Manual Therapy)	33.10 (5.07)	26.65 (4.85)	29.45 (4.66)
Group 3 (Control)	34.45 (4.53)	30.55 (4.33)	30.55 (4.33)
*p*-Value (Interaction)	<0.001		
*p*-Value (between groups)	*p* ^a,b,c^ > 0.05	*p* ^a,b,c,^*	*p* ^a,b,^*
**BPS score (SD)**			
Group 1 (Combined Therapy)	7.85 (1.98)	3.85 (1.08)	3.75 (1.33)
Group 2 (Manual Therapy)	7.45 (1.39)	4.50 (1.35)	5.65 (1.53)
Group 3 (Control)	6.95 (1.95)	6.05 (1.79)	6.05 (1.80)
*p*-Value (Interaction)	<0.001		
*p*-Value (between groups)	*p* ^a,b,c^ > 0.05	*p* ^b,c,^*	*p* ^a,b,^*
**L4-L5 paraspinal intervertebral space PPT, N/cm^2^ Right**			
Group 1 (Combined Therapy)	37.65 (8.23)	60.24 (13.17)	56.22 (12.14)
Group 2 (Manual Therapy)	33.30 (10.54)	45.95 (14.55)	44.20 (14.12)
Group 3 (Control)	31.84 (8.03)	33.44 (8.96)	33.43 (9.11)
*p*-Value (Interaction)	<0.001		
*p*-Value (between groups)	*p* ^a,b,c^ > 0.05	*p* ^a,b,c,^*	*p* ^a,b,c,^*
**L4-L5 paraspinal intervertebral space PPT, N/cm^2^ Left**			
Group 1 (Combined Therapy)	35.59 (6.54)	57.31 (10.52)	53.97 (9.76)
Group 2 (Manual Therapy)	32.26 (10.15)	45.28 (14.36)	44.19 (14.42)
Group 3 (Control)	31.68 (7.46)	33.26 (8.05))	32.06 (8.12)
*p*-Value (Interaction)	<0.001		
*p*-Value (between groups)	*p* ^a,b,c^ > 0.05	*p* ^a,b,c,^*	*p* ^a,b,c,^*
**Quadratus lumborum muscle PPT, N/cm^2^ Right**			
Group 1 (Combined Therapy)	28.15 (6.29)	50.79 (9.94)	49.78 (9.74)
Group 2 (Manual Therapy)	28.96 (8.12)	39.97 (11.21)	34.37 (9.64)
Group 3 (Control)	25.80 (6.38)	26.31 (6.51)	24.21 (5.99)
*p*-Value (Interaction)	<0.001		
*p*-Value (between groups)	*p* ^a,b,c^ > 0.05	*p* ^a,b,c,^*	*p* ^a,b,c,^*
**Quadratus lumborum muscle PPT, N/cm^2^ Left**			
Group 1 (Combined Therapy)	33.39 (7.27)	60.19 (11.70)	58.99 (11.47)
Group 2 (Manual Therapy)	32.37 (11.82)	46.29 (16.90)	38.88 (14.19)
Group 3 (Control)	27.15 (9.83)	28.78 (10.42)	26.76 (9.69)
*p*-Value (Interaction)	<0.001		
*p*-Value (between groups)	*p* ^a,b,c^ > 0.05	*p* ^a,b,c,^*	*p* ^a,b,c,^*

Between group comparison: a = Group 1 versus Group 2; b = Group 1 versus Group 3; c = Group 2 versus Group 3. * Between groups significant comparisons in the post-hoc testing.

## Data Availability

The datasets generated and analyzed during the current study are available from the corresponding author upon reasonable request.

## References

[1] Hartvigsen J., Hancock M.J., Kongsted A., Louw Q., Ferreira M.L., Genevay S., Hoy D., Karppinen J., Pransky G., Sieper J. (2018). What low back pain is and why we need to pay attention. Lancet.

[2] Thiese M.S., Hegmann K.T., Wood E.M., Garg A., Moore J.S., Kapellusch J., Foster J., Ott U. (2014). Prevalence of low back pain by anatomic location and intensity in an occupational population. BMC Musculoskelet. Disord..

[3] Ferreira M.L., de Luca K., Haile L.M., Steinmetz J.D., Culbreth G.T., Cross M., Kopec J.A., Ferreira P.H., Blyth F.M., Buchbinder R. (2023). Global, regional, and national burden of low back pain, 1990–2020, its attributable risk factors, and projections to 2050: A systematic analysis of the Global Burden of Disease Study 2021. Lancet Rheumatol..

[4] O’Sullivan P. (2012). It’s time for change with the management of non-specific chronic low back pain. Br. J. Sports Med..

[5] Perrot S., Cohen M., Barke A., Korwisi B., Rief W., Treede R.D., IASP Taskforce for the Classification of Chronic Pain (2019). The IASP classification of chronic pain for ICD-11: Chronic secondary musculoskeletal pain. Pain.

[6] George S.Z., Fritz J.M., Silfies S.P., Schneider M.J., Beneciuk J.M., Lentz T.A., Gilliam J.R., Hendren S., Norman K.S. (2021). Interventions for the Management of Acute and Chronic Low Back Pain: Revision 2021. J. Orthop. Sports Phys. Ther..

[7] Corp N., Mansell G., Stynes S., Wynne-Jones G., Mørso L., Hill J.C., van der Windt D.A. (2021). Evidence-based treatment recommendations for neck and low back pain across Europe: A systematic review of guidelines. Eur. J. Pain.

[8] Zaina F., Côté P., Cancelliere C., Felice F.D., Donzelli S., Rauch A., Verville L., Negrini S., Nordin M. (2023). A Systematic Review of Clinical Practice Guidelines for Persons With Non-specific Low Back Pain With and Without Radiculopathy: Identification of Best Evidence for Rehabilitation to Develop the WHO’s Package of Interventions for Rehabilitation. Arch. Phys. Med. Rehabil..

[9] Puentedura E.J., Flynn T. (2016). Combining manual therapy with pain neuroscience education in the treatment of chronic low back pain: A narrative review of the literature. Physiother. Theory Pract..

[10] Louw A., Zimney K., Puentedura E.J., Diener I. (2016). The efficacy of pain neuroscience education on musculoskeletal pain: A systematic review of the literature. Physiother. Theory Pract..

[11] Watson J.A., Ryan C.G., Cooper L., Ellington D., Whittle R., Lavender M., Dixon J., Atkinson G., Cooper K., Martin D.J. (2019). Pain Neuroscience Education for Adults With Chronic Musculoskeletal Pain: A Mixed-Methods Systematic Review and Meta-Analysis. J. Pain.

[12] Saracoglu I., Arik M.I., Afsar E., Gokpinar H.H. (2022). The effectiveness of pain neuroscience education combined with manual therapy and home exercise for chronic low back pain: A single-blind randomized controlled trial. Physiother. Theory Pract..

[13] Song J., Kim H., Jung J., Lee S. (2023). Soft-Tissue Mobilization and Pain Neuroscience Education for Chronic Nonspecific Low Back Pain with Central Sensitization: A Prospective Randomized Single-Blind Controlled Trial. Biomedicines.

[14] Aanesen F., Berg R., Løchting I., Tingulstad A., Eik H., Storheim K., Grotle M., Øiestad B.E. (2021). Motivational Interviewing and Return to Work for People with Musculoskeletal Disorders: A Systematic Mapping Review. J. Occup. Rehabil..

[15] Alperstein D., Sharpe L. (2016). The efficacy of motivational interviewing in adults with chronic pain: A meta-analysis and systematic review. J. Pain.

[16] Nijs J., Wijma A.J., Willaert W., Huysmans E., Mintken P., Smeets R., Goossens M., van Wilgen C.P., Van Bogaert W., Louw A. (2020). Integrating motivational interviewing in pain neuroscience education for people with chronic pain: A practical guide for clinicians. Phys. Ther..

[17] Urbaniak G.C., Plous S. (2013). Research Randomizer (Version 4.0). https://www.randomizer.org/.

[18] Childs J.D., Piva S.R., Fritz J.M. (2005). Responsiveness of the numeric pain rating scale in patients with low back pain. Spine.

[19] Chiarotto A., Maxwell L.J., Ostelo R.W., Boers M., Tugwell P., Terwee C.B. (2019). Measurement properties of Visual Analogue Scale, Numeric Rating Scale, and Pain Severity Subscale of the Brief Pain Inventory in Patients with Low Back Pain: A Systematic Review. J. Pain.

[20] Ostelo R.W.J.G., de Vet H.C.W. (2005). Clinically important outcomes in low back pain. Best. Pract. Res. Clin. Rheumatol..

[21] Jenks A., Hoekstra T., van Tulder M., Ostelo R.W., Rubinstein S.M., Chiarotto A. (2022). Roland-Morris Disability Questionnaire, Oswestry Disability Index, and Quebec Back Pain Disability Scale: Which has superior measurement properties in older adults with Low Back Pain?. J. Orthop. Sports Phys. Ther..

[22] Roland M., Fairbank J. (2000). The Roland-Morris disability questionnaire and the Oswestry disability questionnaire. Spine.

[23] Boscainos P.J., Sapkas G., Stilianessi E., Prouskas K., Papadakis S.A. (2003). Greek versions of the Oswestry and Roland-Morris Disability Questionnaires. Clin. Orthop. Relat. Res..

[24] Imamura M., Alfieri F.M., Filippo T.R.M., Battistella L.R. (2016). Pressure pain thresholds in patients with chronic nonspecific low back pain. J. Back. Musculoskelet. Rehabil..

[25] Imamura M., Chen J., Matsubayashi S.R., Targino R.A., Alfieri F.M., Bueno D.K., Hsing W.T. (2013). Changes in pressure pain threshold in patients with chronic nonspecific low back pain. Spine.

[26] Balaguier R., Madeleine P., Vuillerme N. (2016). Intra-session absolute and relative reliability of pressure pain thresholds in the low back region of vine-workers: Ffect of the number of trials. BMC Musculoskelet. Disord..

[27] Voogt L., de Vries J., Meeus M., Struyf F., Meuffels D., Nijs J. (2015). Analgesic effects of manual therapy in patients with musculoskeletal pain: A systematic review. Man. Ther..

[28] Liu H., Huang L., Yang Z., Li H., Wang Z., Peng L. (2021). Fear of movement/(Re) injury: An update to descriptive review of the related measures. Front. Psychol..

[29] French D.J., France C.R., Vigneau F., French J.A., Evans R.T. (2007). Fear of movement/(re)injury in chronic pain: A psychometric assessment of the original English version of the Tampa scale for kinesiophobia (TSK). Pain.

[30] Georgoudis G., Raptis K., Koutserimpas C. (2022). Cognitive Assessment of Musculoskeletal Pain: Validity and Reliability of the Greek Version of the Tampa Scale of Kinesiophobia in Patients Suffering from Chronic Low Back Pain. Maedica.

[31] Dupuis F., Cherif A., Batcho C., Massé-Alarie H., Roy J.S. (2023). The Tampa Scale of Kinesiophobia: A Systematic Review of its psychometric properties in people with musculoskeletal pain. Clin. J. Pain.

[32] Sullivan M.J.L., Bishop S.R., Pivik J.R. (1995). The Pain Catastrophizing Scale: Development and Validation. Psychol. Assess..

[33] Quartana P.J., Campbell C.M., Edwards R.R. (2009). Pain catastrophizing a critical review. Expert Rev. Neurother..

[34] Ogunlana M.O., Odole A.C., Adejumo A., Odunaiya N. (2015). Catastrophising, pain, and disability in patients with nonspecific low back pain. Hong Kong Physiother. J..

[35] Christakou A. (2021). Cross-cultural adaptation of the Pain Catastrophizing Scale in Greek clinical population. Hong Kong Physiother. J..

[36] Strand L.I., Moe-Nilssen R., Ljunggren A.E. (2002). Back performance scale for the assessment of mobility-related activities in people with back pain. Phys. Ther..

[37] Magnussen L., Strand L.I., Lygren H. (2004). Reliability and Validity of the Back Performance Scale: Observing Activity Limitation in Patients with Back Pain. Spine.

[38] Louw A., Nijs J., Puentedura E.J. (2017). A clinical perspective on a pain neuroscience education approach to manual therapy. J. Man. Manip. Ther..

[39] Louw A., Zimney K., O’Hotto C., Hilton S. (2016). The clinical application of teaching people about pain. Physiother. Theory Pract..

[40] RetrainPain. https://www.retrainpain.org/languages/greek.

[41] Cohen J. (1988). Statistical Power Analysis for the Behavioral Sciences.

[42] Saracoglu I., Arik M.I., Afsar E., Gokpinar H.H. (2020). The short-term effects of neuroscience pain education on quality of life in patients with chronic low back pain: A single-blinded randomized controlled trial. Eur. J. Integr. Med..

[43] Tavares F.A.G., Rossiter J.V.A., Lima G.C.L., de Oliveira L.G., Cavalcante W.S., Avila M.A., George S.Z., Chaves T.C. (2023). Additional effect of pain neuroscience education to spinal manipulative therapy on pain and disability for patients with chronic low back pain: A randomized controlled trial. Braz. J. Phys. Ther..

[44] Wittink H., Oosterhaven J. (2018). Patient education and health literacy. Musculoskelet. Sci. Pract..

[45] Malfliet A., Kregel J., Coppieters I., De Pauw R., Meeus M., Roussel N., Cagnie B., Danneels L., Nijs J. (2018). Effect of pain neuroscience education combined with cognition-targeted motor control training on chronic spinal pain a randomized clinical trial. JAMA Neurol..

[46] Bodes Pardo G., Lluch Girbés E., Roussel N.A., Izquierdo T.G., Penick V.J., Martín D.P. (2018). Pain Neurophysiology Education and Therapeutic Exercise for Patients With Chronic Low Back Pain: A Single-Blind Randomized Controlled Trial. Arch. Phys. Med. Rehabil..

[47] Bialosky J.E., Beneciuk J.M., Bishop M.D., Coronado R.A., Penza C.W., Simon C.B., George S.Z. (2018). Unraveling the Mechanisms of Manual Therapy: Modeling an Approach. J. Orthop. Sports Phys. Ther..

[48] Kamonseki D.H., Christenson P., Rezvanifar S.C., Calixtre L.B. (2021). Effects of manual therapy on fear avoidance, kinesiophobia and pain catastrophizing in individuals with chronic musculoskeletal pain: Systematic review and meta-analysis. Musculoskelet. Sci. Pract..

[49] Ramós-Martín G.J., Rodríguez-Nogueira O. (2021). Effectiveness of pain neuroscience education alone or combined with therapeutic exercise in chronic low back pain patients: A systematic review. Fisioterapia.

[50] Leeuw M., Goossens M.E.J.B., Linton S.J., Crombez G., Boersma K., Vlaeyen J.W.S. (2007). The fear-avoidance model of musculoskeletal pain: Current state of scientific evidence. J. Behav. Med..

